# Potential impact of a nonavalent HPV vaccine on the occurrence of HPV-related diseases in France

**DOI:** 10.1186/s12889-015-1779-1

**Published:** 2015-05-02

**Authors:** Didier Riethmuller, Anne-Carole Jacquard, Jean Lacau St Guily, François Aubin, Xavier Carcopino, Pierre Pradat, André Dahlab, Jean-Luc Prétet

**Affiliations:** Service de gynécologie obstétrique, CHU Saint Jacques, Besançon, France; Sanofi Pasteur MSD, 12 rue Jonas Salk, 69367 Lyon, Cedex 07 France; Service d’Oto-rhino-laryngologie et chirurgie cervico-faciale, Hôpital Tenon, Université de Paris 6 et Faculté de Pierre et Marie Curie, Assistance Publique-Hôpitaux de Paris, Paris, France; Service de dermatologie, Centre Hospitalier Universitaire Saint-Jacques, Besançon, France; Department of Obstetrics and Gynaecology, Hôpital Nord, Assistance Publique des Hôpitaux de Marseille (APHM), Aix-Marseille Université (AMU), CNRS, IRD, Avignon Université, IMBE UMR 7263, 13397 Marseille, France; Department of Hepatology, Hôpital de la Croix-Rousse, Hospices Civils de Lyon, Lyon, France; Université Franche-Comte, F-25000 Besançon, France; EA 3181, FED4234, LabEx LipSTIC ANR-11-LABX-0021, CIC-BT 506, F-25000 Besancon, France; CHRU Besançon, F-25000 Besançon, France

**Keywords:** HPV, Human papillomavirus, Vaccine, Invasive cervical cancer, Cervical intraepithelial neoplasia, Genital warts, Anal cancer

## Abstract

**Background:**

Human Papillomavirus (HPV) infection is known to be associated with a number of conditions including cervical, vaginal, vulvar, penile, anal neoplasias and cancers, oropharynx cancers and genitals warts (GW). Two prophylactic vaccines are currently available: a bivalent vaccine designed to prevent HPV type 16 and 18 infection and a quadrivalent vaccine targeting HPV 6, 11, 16, and 18. In France, HPV vaccination is recommended in 11-14 year-old girls with a catch-up for girls aged 15-19. The objective of this study was to assess the potential impact of an HPV 6/11/16/18/31/33/45/52/58 nonavalent vaccine on anogenital and oropharyngeal HPV-related diseases in France.

**Methods:**

HPV genotype distributions from 6 multicentric retrospective studies (EDiTH I to VI) were analyzed including 516 cases of invasive cervical cancers (ICC), 493 high-grade cervical neoplasias (CIN2/3), 397 low-grade squamous intraepithelial lesions (LSIL), 423 GW, 366 anal cancer and 314 oropharyngeal carcinomas. Low and high estimates of HPV vaccine impact were calculated as follows: low estimate: prevalence of HPV 6/11/16/18/31/33/45/52/58 genotypes alone or in association but excluding presence of another HPV type; high estimate: prevalence of HPV 6/11/16/18/31/33/45/52/58 genotypes alone or in association, possibly in presence of another HPV type.

**Results:**

Estimates of potential impact varied from 85% (low estimate) to 92% (high estimate) for ICC, 77% to 90% for CIN2/3, 26% to 56% for LSIL, 69% to 90% for GW, 81% to 93% for anal cancer, and 41% to 44% for oropharyngeal carcinomas. Compared to the quadrivalent vaccine, the proportion of additional cases potentially prevented by the nonavalent vaccine was 9.9%-15.3% for ICC, 24.7%-33.3% for CIN2/3, 12.3%-22.7% for LSIL, 2.1%-5.4% for GW, 8.5%-10.4% for anal cancer, and 0.0%-1.6% for oropharyngeal carcinoma.

**Conclusions:**

The nonavalent HPV vaccine showed significant increased potential impact compared to the HPV 6/11/16/18 quadrivalent vaccine for ICC, CIN2/3 and LSIL. Considering a 100% vaccine efficacy and high vaccine coverage, about 90% of ICC, CIN2/3, GW or anal cancer cases could be prevented by a nonavalent HPV vaccine in France.

**Electronic supplementary material:**

The online version of this article (doi:10.1186/s12889-015-1779-1) contains supplementary material, which is available to authorized users.

## Background

Human Papillomavirus (HPV) infection is known to be associated with a number of conditions including cervical, vaginal, vulvar, penile, anal neoplasias and cancers, oropharynx cancers and genitals warts (GW) [1-5]. Two prophylactic vaccines using L1 virus-like particles (VLP) are available and widely marketed internationally. These vaccines were primarily developed with the aim of reducing HPV-related cervical cancer. The first one (Cervarix®) is a bivalent vaccine designed to prevent high-risk HPV type 16 and 18 infection. The second one (Gardasil®), is a quadrivalent vaccine targeting HPV 6, 11, 16, and 18 [6], HPV low-risk types 6 and 11 being associated with 90% of GW [7,8]. In France, HPV vaccination is recommended in 11-14 year-old girls with a catch-up for girls aged 15-19 [9].

Worldwide studies in invasive cervical cancer (ICC) cases reported that the most commonly encountered HPV types after HPV 16 and 18 were HPV 31, 33, 35, 45, 52, and 58 [10,11]. Merck has been developing a nonavalent vaccine targeting five additional high-risk HPV types (HPV 31/33/45/52/58) to the HPV types 6/11/16/18 contained in the quadrivalent vaccine [12]. Serrano et al. estimated that the addition of HPV 31/33/45/52/58 to HPV types included in current vaccines could prevent almost 90% of ICC cases worldwide [13].

The French EDiTH studies (Etude de la Distribution des Types d’HPV) reported the HPV genotype distribution in invasive cervical cancer (EDiTH I) [14], high-grade cervical neoplasias (CIN2/3) (EDiTH II) [15], low-grade squamous intraepithelial lesions (LSIL) (EDiTH III) [16], external acuminata condylomata (genital warts) (EDiTH IV) [17], anal cancer (EDiTH V) [18] and oropharyngeal cancer (EDiTH VI) [19]. In these studies, the estimated potential impact of a 6/11/16/18 quadrivalent vaccine varied from 14-33% in LSIL to about 70-83% in cervical or anal cancers.

These studies have shown that a proportion of HPV-related lesions were not targeted by currently available vaccines. The objective of the present study was thus to assess the potential impact in France of a 6/11/16/18/31/33/45/52/58 nonavalent HPV vaccine on anogenital and oropharyngeal HPV-related diseases, and to compare this impact with the 6/11/16/18 quadrivalent vaccine.

## Methods

### Studies

We reanalyzed data from 6 multicenter retrospective studies (EDiTH I to EDiTH VI) published elsewhere [14-19]. For each study, details regarding patients’ data, histological specimens’ inclusion/exclusion criteria, DNA isolation, HPV genotyping and ethical considerations can be found in the respective publications.

### Statistical analysis

Histological specimens of 516 invasive cervical cancers (HPV prevalence, 97.1%), 493 high grade cervical neoplasias (HPV prevalence, 98.2%), 397 low-grade squamous intraepithelial lesions (HPV prevalence, 98.2%), 423 external acuminata condylomata (HPV prevalence, 98.8%), 366 anal cancer (HPV prevalence, 96.7%) and 314 oropharyngeal carcinomas (HPV prevalence, 46.5%) were included in the analysis.

For each condition, HPV genotype distributions were used to assess the potential impact of the quadrivalent and the nonavalent vaccines in France. A low and a high estimate of the vaccine impact were calculated as follows. For the quadrivalent vaccine, low estimate was the prevalence of HPV 6/11/16/18 genotypes alone or in association but excluding presence of another HPV type; high estimate was the prevalence of HPV 6/11/16/18 genotypes alone or in association possibly in presence of another HPV type. For the nonavalent vaccine, low estimate was the prevalence of HPV 6/11/16/18/31/33/45/52/58 genotypes alone or in association but excluding presence of another HPV type; high estimate was the prevalence of HPV 6/11/16/18/31/33/45/52/58 genotypes alone or in association possibly in presence of another HPV type. Additional file 1: Table S1 presents genotypes and combination of genotypes used to define the low and high estimates for the quadrivalent and nonavalent vaccines. Estimates are presented with their 95% confidence intervals calculated based on the cumulative binomial distribution.

The absolute additional potential impact of the nonavalent vaccine, i.e. the proportion of additional cases potentially prevented by the nonavalent vaccine compared to the quadrivalent vaccine, was calculated as follows:

$$ \frac{n_{nonavalent}-{n}_{quadrivalent}}{N} \times 100 $$

with *n* being the number of cancer cases potentially prevented and *N* the total number of cancer cases.

The relative additional potential impact of the nonavalent vaccine compared to the quadrivalent vaccine was calculated as follows:

$$ \frac{n_{nonavalent}-{n}_{quadrivalent}}{n_{quadrivalent}} \times 100 $$

with *n* representing the number of potentially prevented cancer cases.

## Results

HPV genotypes in the six EDiTH studies are described in details in Table 1. Single infections by HPV 6/11/16/18/31/33/45/52/58 were found in 362 (70.2%), 299 (60.6%), 84 (21.2%), 251 (59.3%), 257 (70.2%) and 130 (41.4%) of invasive cervical cancers, high-grade cervical neoplasias, low-grade squamous intraepithelial lesions, external acuminata condylomata, anal cancer and oropharyngeal carcinomas, respectively. Corresponding multiple infections by HPV 6/11/16/18/31/33/45/52/58, excluding any other HPV genotype, were found in 74 (14.3%), 79 (16.0%), 19 (4.8%), 39 (9.2%), 39 (10.7%) and 0 (0,0%), and corresponding multiple infections by HPV 6/11/16/18/31/33/45/52/58, with or without another HPV genotype, in 113 (21,9%), 142 (28,8%), 139 (35,0%), 131 (31,0%), 84 (23,0%) and 8 (2,5%).

**Table 1:** **HPV genotype distribution in the different EDiTH studies**

	**Invasive cervical cancers**	**High-grade cervical neoplasias**	**Low-grade squamous intraepithelial lesions**	**External acuminata condylomata**	**Anal cancer**	**Oropharyngeal carcinomas**
	**n**	**n**	**n**	**n**	**n**	**n**
HPV (+)	501 (97.1)	484 (98.2)	390 (98.2)	418 (98.8)	354 (96.7)	146 (46.5)
HPV (-)	15 (2.9)	9 (1.8)	7 (1.8)	5 (1.2)	12 (3.3)	168 (53.5)
**Total**	516 (100)	493 (100)	397 (100)	423 (100)	366 (100)	314 (100)
**Total single infections**	386 (74.8)	333 (67.5)	192 (48.4)	281 (66.4)	265 (72.4)	138 (43.9)
Total single infections 6/11/16/18	316 (61.2)	203 (41.2)	50 (12.6)	248 (58.6)	234 (63.9)	130 (41.4)
Single infections 6	1 (0.2)	0 (0.0)	11 (2.8)	195 (46.1)	13 (3.6)	0 (0.0)
Single infections 11	1 (0.2)	1 (0.2)	0 (0.0)	42 (9.9)	4 (1.1)	0 (0.0)
Single infections 16	275 (53.3)	199 (40.4)	31 (7.8)	11 (2.6)	216 (59.0)	130 (41.4)
Single infections 18	39 (7.6)	3 (0.6)	8 (2.0)	0 (0.0)	1 (0.3)	0 (0.0)
Total single infections 31/33/45/52/58	46 (8.9)	96 (19.5)	34 (8.6)	3 (0.7)	23 (6.3)	0 (0.0)
Single infections 31	17 (3.3)	41 (8.3)	8 (2.0)	1 (0.2)	4 (1.1)	0 (0.0)
Single infections 33	11 (2.1)	27 (5.5)	1 (0.3)	0 (0.0)	11 (3.0)	0 (0.0)
Single infections 45	9 (1.7)	0 (0.0)	6 (1.5)	0 (0.0)	0 (0.0)	0 (0.0)
Single infections 52	6 (1.2)	13 (2.6)	8 (2.0)	1 (0.2)	5 (1.4)	0 (0.0)
Single infections 58	3 (0.6)	15 (3.0)	11 (2.8)	1 (0.2)	3 (0.8)	0 (0.0)
Total single infections 6/11/16/18/31/33/45/52/58	362 (70.2)	299 (60.6)	84 (21.2)	251 (59.3)	257 (70.2)	130 (41.4)
Single infections others	24 (4.7)	34 (6.9)	108 (27.2)	30 (7.1)	8 (2.2)	8 (2.5)
**Total multiple infections**	115 (22.3)	151 (30.6)	198 (49.9)	137 (32.4)	89 (24.3)	8 (2.5)
Multiple infections 6/11/16/18 excluding any other HPV genotypes	41 (7.9)	11 (2.2)	4 (1.0)	19 (4.5)	24 (6.6)	0 (0.0)
Multiple infections 6/11/16/18 with or without another HPV genotype	108 (20.9)	116 (23.5)	83 (20.9)	125 (29.6)	76 (20.8)	3 (1.0)
Multiple infections 6/11/16/18/31/33/45/52/58 excluding any other HPV genotypes	74 (14.3)	79 (16.0)	19 (4.8)	39 (9.2)	39 (10.7)	0 (0.0)
Multiple infections 6/11/16/18/31/33/45/52/58 with or without another HPV genotype	113 (21.9)	142 (28.8)	139 (35.0)	131 (31.0)	84 (23.0)	8 (2.5)
Multiple infections other HPV excluding 6/11/16/18/31/33/45/52/58	2 (0.4)	9 (1.8)	59 (14.9)	6 (1.4)	5 (1.4)	0 (0.0)

**Figure 1:**
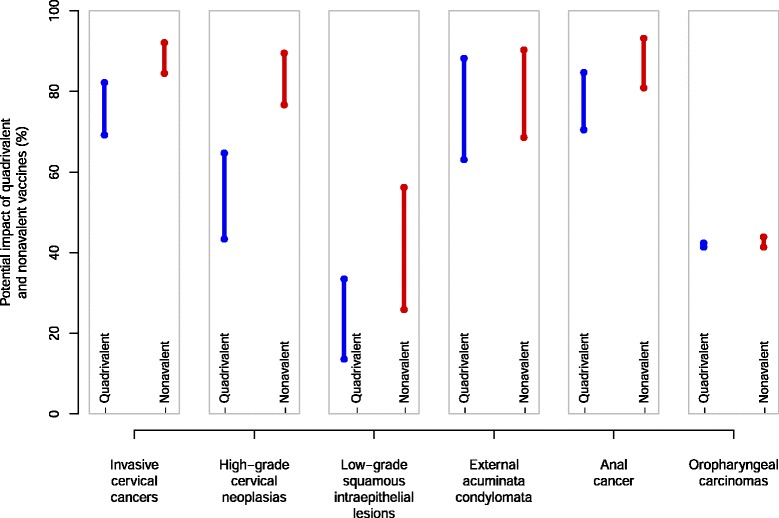
Potential impact of quadrivalent and nonavalent HPV vaccines on invasive cervical cancers, high-grade cervical neoplasias, low-grade squamous intraepithelial lesions, external acuminata condylomata, anal cancers, and oropharyngeal carcinomas in France.

The potential impact of the quadrivalent and nonavalent HPV vaccines assessed by low and high estimates is presented in Figure 1. The nonavalent HPV vaccine showed increased impact compared to the quadrivalent vaccine for invasive cervical cancers, high-grade cervical neoplasias and low-grade squamous intraepithelial lesions. The number of genotypes targeted by the nonavalent vaccine varied between 84.5% (low estimate, 95%CI 81.0 to 87.8) and 92.1% (high estimate, 95%CI 89.5 to 94.3) for invasive cervical cancers, between 76.7% (95%CI 72.2 to 81.0) and 89.5% (95%CI 86.4 to 92.3) for high-grade cervical neoplasias, and between 25.9% (95%CI 17.5 to 35.0) and 56.2% (95%CI 49.8 to 62.8) for low-grade squamous intraepithelial lesions.

The absolute additional impact of the nonavalent vaccine (i.e the number of additional cases that could be prevented by the nonavalent vaccine) as well as the relative additional impact (i.e compared to the quadrivalent vaccine) are presented in Table 2. Again, limited impact was found for the nonavalent vaccine compared to the quadrivalent one for external acuminata condylomata, anal cancer and oropharyngeal carcinomas. The absolute additional impact of the nonavalent vaccine lied between 9.9% and 15.3% for invasive cervical cancers, between 24.7% and 33.3% for high-grade cervical neoplasias, and between 12.3% and 22.7% for low-grade squamous intraepithelial lesions. The benefit of the nonavalent vaccine compared to the quadrivalent vaccine ranged between 12.0% and 22.1% for invasive cervical cancers, between 38.2% and 76.6% for high grade cervical neoplasias, and between 67.7% and 90.7% for low-grade squamous intraepithelial lesions.

**Table 2:** **Overall proportion (low and high estimates) of cases targeted by a quadrivalent or a nonavalent HPV vaccine**

		**Low estimate**	**High estimate**
Invasive cervical cancers (EDITH I)	Quadrivalent	69.2 [64.4-73.9]	82.2 [78.5-85.8]
	Nonavalent	84.5 [81.0-87.8]	92.1 [89.5-94.3]
	Absolute additional Impact* (% of additional prevented cases)	15.3% (p < 0.001)	9.9% (p < 0.001)
	Relative additional Impact**	22.1%	12.0%
High-grade cervical neoplasias (EDITH II)	Quadrivalent	43.4 [36.9-50.0]	64.7 [59.6-69.9]
	Nonavalent	76.7 [72.2-81.0]	89.5 [86.4-92.3]
	Absolute additional Impact (% of additional prevented cases)	33.3% (p < 0.001)	24.7% (p < 0.001)
	Relative additional Impact	76.6%	38.2%
Low-grade squamous intraepithelial lesions (EDITH III)	Quadrivalent	13.6 [5.6-24.1]	33.5 [25.6-41.4]
	Nonavalent	25.9 [17.5-35.0]	56.2 [49.8-62.8]
	Absolute additional Impact (% of additional prevented cases)	12.3% (p < 0.001)	22.7% (p < 0.001)
	Relative additional Impact	90.7%	67.7%
External acuminata condylomata (EDITH IV)	Quadrivalent	63.1 [57.3-68.9]	88.2 [84.7-91.4]
	Nonavalent	68.6 [63.1-73.8]	90.3 [87.2-93.2]
	Absolute additional Impact (% of additional prevented cases)	5.4% (p = 0.095)	2.1% (p = 0.318)
	Relative additional Impact	8.6%	2.4%
Anal cancers (EDITH V)	Quadrivalent	70.5 [64.7-76.0]	84.7 [80.6-88.7]
	Nonavalent	80.9 [76.4-85.1]	93.2 [90.3-95.6]
	Absolute additional Impact (% of additional prevented cases)	10.4% (p = 0.001)	8.5% (p < 0.001)
	Relative additional Impact	14.7%	10.0%
Oropharyngeal carcinomas (EDITH VI)	Quadrivalent	41.4 [33.1-50.0]	42.4 [33.8-51.1]
	Nonavalent	41.4 [33.1-50.0]	43.9 [35.5-52.2]
	Absolute additional Impact (% of additional prevented cases)	0.0% (NC)	1.6% (p = 0.687)
	Relative additional Impact	0.0%	3.8%

*Absolute additional potential impact of the nonavalent vaccine.

**Relative additional potential impact of the nonavalent vaccine.

NC, not calculated.

## Discussion

Based on large number of cases, the national multicentre EDiTH studies previously reported the HPV genotype distribution for different cervical, anal, or oropharyngeal diseases in France [14-20]. Assuming a 100% vaccine efficacy and vaccination coverage, the EDiTH results suggested that a 6/11/16/18 quadrivalent HPV vaccine could prevent between 70% and 83% of ICC and anal cancer cases.

The attribution of cases to HPV types is often complicated by the existence of multiple infections characterized by the presence of several HPV types in the same tumor. Therefore, the potential benefit of an HPV vaccine is difficult to assess especially when HPV types not targeted by the vaccine are present. In all EDiTH studies and in the present study as well, we thus calculated a low and a high estimate of the vaccine impact based on presence of single or multiple infections. It is possible that the high estimate gives an overestimation of the potential impact of the vaccine since one assumes that HPV types targeted by the vaccine are causally related to the lesion in which they are found even in the presence of another HPV type. It is thus reasonable to believe that the “true” potential impact lies somewhere between the low and the high estimates.

If low estimate calculations are considered, the present study indicates that the absolute additional impact of a nonavalent vaccine is highest for CIN2/3 with a 33% increase in the proportion of cases targeted by the nonavalent vaccine. This additional benefit is intermediate for ICC (15% increase), LSIL (12%) and anal cancer (10%) whereas almost no additional benefit is observed for genital warts (5%) and oropharyngeal carcinomas (0%). The EDiTH II study showed that between 43% and 65% of CIN2/3 cases were associated with HPV 6/11/16/18 (low and high estimates) [15] whereas the present study indicates that a nonavalent vaccine could target 77% and up to 90% of all CIN2/3 cases. This benefit on the prevention of CIN2/3 cases could have a real public health impact by reducing the costs related to the management of these lesions. It is indeed estimated that 25,000 to 30,000 conizations are performed in France annually [21]. The benefit on ICC would also be substantial with up to 92% of ICC cases that could be targeted by a nonavalent vaccine. Even if the proportion of LSIL cases attributed to HPV 6/11/16/18 was rather low (14-34%), the proportion of cases associated with HPV types targeted by the nonavalent vaccine is increased by almost 90% (14% vs 26% considering the low estimates). However, for genital warts, only about 5% of cases are attributed to the additional HPV types found in the nonavalent vaccine (HPV 31/33/45/52/58) resulting in a low efficacy benefit. Similarly, no oropharyngeal carcinoma case was associated with these additional HPV types suggesting no additional benefit of a nonavalent vaccine in this group. This limited additional benefit on oropharyngeal carcinoma and genital warts is mainly explained by the fact that these conditions are almost exclusively associated with HPV types targeted by the bivalent or quadrivalent vaccines. Anal cancers are known to be mainly associated with HPV 16. A possible explanation for the observed additional benefit of the nonavalent vaccine (15%) is that we included some HIV positive patients (14%) with higher risk of multiple infection (50% vs 20% in HIV negative patients).

It should be noticed that a strong epidemiological impact characterized by significant reductions in HPV-related precancerous lesions and cancers may be achieved only if vaccination coverage reaches more than 80% [22]. However, by the end of 2013, the vaccination coverage with 3 doses reached only 20% of 16 year-old women in France [23]. By raising public awareness of the importance of HPV vaccination, general practitioners and gynecologists have to play an important role for increasing vaccination coverage.

A possible limitation of the present study is that HPV positivity in the EDiTH studies was based on HPV DNA detection only which could have resulted in a slight overestimation of the proportion of cancers potentially attributed to HPV. This is particularly true for oropharyngeal carcinomas for which HPV RNA detection would be a more accurate marker for those related to HPV infection. Moreover, other risk factors such as smoking or alcohol abuse are possibly involved. We calculated low and high estimates with the aim of taking into account the lack of knowledge regarding the causal relationship between each lesion and multiple HPV infection types. Low estimates suppose that the vaccine only prevents cases with genotypes targeted by the vaccine, while high estimates rather suppose that the vaccine prevents all cases where at least one genotype targeted by the vaccine is present, even in the presence of another genotype. This assumes that the other genotype is not involved in the occurrence of the lesion. Of course, true effects are in-between and it should be noted that alternative methods based on proportional (i.e., weighted) or hierarchical attributions of genotypes to disease categories have been proposed, providing intermediate estimates [10,24,25]. We nevertheless preferred to report estimates intervals rather that single estimates.

However, our results are in accordance with previous results reporting that a nonavalent vaccine would increase the protection from 70% to almost 90% of the infections responsible for ICC [13]. Moreover, a model-based analysis showed that at the population level, the switch from a bivalent or a quadrivalent to a nonavalent vaccine would further reduce the occurrence of precancerous lesions and cervical cancer [26].

In France, both the bivalent and the quadrivalent vaccines are available with a predominant use of the quadrivalent [27]. Gardasil is currently indicated for the prevention of premalignant genital lesions (cervical, vulvar and vaginal), premalignant anal lesions, cervical cancers, anal cancers, and genital warts (condyloma acuminata) causally related to specific HPV types [28]. It should thus be borne in mind that the potential vaccine impact we assessed is hypothetical and concerns some outcomes (e.g. oropharyngeal carcinoma) for which no specific indication exists yet.

Pap smear screening is and will remain a very efficient tool for the prevention of ICC. Even if screening of elderly women should still be highly recommended, HPV vaccination could reduce and hinder the spread of the virus and prevent HPV-related diseases and cancers for which no screening strategies are available.

## Conclusion

The nonavalent HPV vaccine showed significant increased potential impact compared to the HPV 6/11/16/18 quadrivalent vaccine for ICC, CIN2/3 and LSIL. Nonavalent vaccination could thus be a cost-effective alternative [29] with almost 90% of ICC, CIN2/3, genital warts and anal cancer cases being potentially prevented.

## Additional file

Additional file 1: Table S1. Definition of low and high estimates.

## Abbreviations

CIN: Cervical intraepithelial neoplasia
GW: Genital warts
HPV: Human papillomavirus
ICC: Invasive cervical cancer
LSIL: Low-grade squamous intraepithelial lesion
