# Supplementary material for: Potential impact of a nonavalent HPV vaccine on the occurrence of HPV-related diseases in France
Source: BMC Public Health. 2015 May 2;15:453. doi: 10.1186/s12889-015-1779-1 (PMC4433025; doi:10.1186/s12889-015-1779-1)
Supplement: Additional file 1: Table S1. — Definition of low and high estimates. [file 12889_2015_1779_MOESM1_ESM.pdf]

Supplementary Table S1: Definition of low and high estimates

	**Low estimate**	**High estimate**
**Quadrivalent vaccine**	Single infections 6	Single infections 6
	Single infections 11	Single infections 11
	Single infections 16	Single infections 16
	Single infections 18	Single infections 18
	Multiple infections 6/11/16/18 *excluding any other HPV type*	Multiple infections 6/11/16/18 with or without another HPV type
**Nonavalent vaccine**	Single infections 6	Single infections 6
	Single infections 11	Single infections 11
	Single infections 16	Single infections 16
	Single infections 18	Single infections 18
	Single infections 31	Single infections 31
	Single infections 33	Single infections 33
	Single infections 45	Single infections 45
	Single infections 52	Single infections 52
	Single infections 58	Single infections 58
	Multiple infections 6/11/16/18/31/33/45/52/58 *excluding any other HPV type*	Multiple infections 6/11/16/18/31/33/45/52/58 with or without another HPV type
